# Source apportionment of VOCs and their impacts on surface ozone in an industry city of Baoji, Northwestern China

**DOI:** 10.1038/s41598-017-10631-4

**Published:** 2017-08-30

**Authors:** Yonggang Xue, Steven Sai Hang Ho, Yu Huang, Bowei Li, Liqin Wang, Wenting Dai, Junji Cao, Shuncheng Lee

**Affiliations:** 1 Key Lab of Aerosol Chemistry & Physics, Institute of Earth Environment, Chinese Academy of Sciences, Xi’an, 710061 China; 2 State Key Lab of Loess and Quaternary Geology (SKLLQG), Institute of Earth Environment, Chinese Academy of Sciences, Xi’an, 710061 China; 3 Division of Atmospheric Sciences, Desert Research Institute, Reno, Nevada USA; 4 School of Human Settlements and Civil Engineering, Xi’an Jiaotong University, Xi’an, 710049 China; 5 Department of Civil and Environmental Engineering, The Hong Kong Polytechnic University, Hung Hom, Hong Kong

## Abstract

Level of surface ozone (O_3_) has been increasing continuously in China in recent years, while its contributors and formation pathways are less understood. In this study, distributions of volatile organic compounds (VOCs) and the roles on O_3_ pollution have been investigated in a typical industrial city of Baoji in Northwestern China by means of monitoring of their concentrations and other trace gases_._ The air samples have been collected at three sites according to urban function area. Concentration of VOCs in Weibin site, which near to industrial zone, was higher than most of other cities in China, and the ambient VOCs were dominated by aromatics and alkenes. The temporal variations of VOCs and O_3_ coincided with the surface wind, implying that the formation of O_3_ was impacted by both exports of plumes upwind and local photochemical reactions. Result of source apportionment indicated that industrial emission, vehicular exhaust, and solvent evaporation were three major pollution origins. Alkenes and aromatics contributed to the largest fractions of photochemical reactivity, suggesting the strong influences from industrial and traffic sectors. The study presents the characteristic VOCs and other factors in the contribution of O_3_ formation in China.

## Introduction

Ozone (O_3_) is important constituent of the atmosphere, the concentration of ozone mostly decreases from stratosphere to surface ground downward^1^. Surface or tropospheric O_3_ originated mainly from regular transportation from stratosphere^2^, direct emissions in source-dominated regions^3^, and photochemical reactions between volatile organic compounds (VOCs) and nitrogen oxides (NO_x_)^4, 5^. In between, photochemistry plays an important role on both sources and sinks of O_3_, subjected to the atmospheric NO_x_ levels^4, 6^. Xue *et al*.^3^ reported that the in-*situ* photochemical production is the main source for the surface O_3_ in megacities in China based on calculations with observation-based model. In urban site, the mixing ratio of NO_x_ is often high (up to 70 ppbv) due to large quantities of emissions from heavy industries and vehicular engines. The O_3_ budget is thus sensitive to photochemical reactivity of VOCs and NO_x_^3, 7–9^. Under short term regulatory measures, reduction of anthropogenic VOCs emission is efficient in shrinking of O_3_ peak. However, the approaches only lead the reaction mechanism between VOCs-NO_x_-O_3_ transferred from NO_x_-dependent to VOCs-dependent while VOCs becomes a limiting factor, in the case of no decline of NO_x_ emission. Instead, to further reduce surface O_3_ level, regulations on both VOCs, biogenic VOCs (BVOCs) and NO_x_ are needed in a long term run^9^.

Understanding of the sources of VOCs is a base for O_3_ pollution control. Common anthropogenic activities include coal burning from industrial and residential uses, vehicle exhausts, gasoline volatilization, solvent use, petrochemical manufacturing and biomass burning^10–17^. In addition, biogenic source, in particular of vegetation emission, is a vital factor in VOCs budget^18–20^. Source apportionments of VOCs in ambient environments are always deployed with receptor models, like positive matrix factorization (PMF), chemical mass balance (CMB), and UNMIX^21–23^. In Northern China (e.g., Beijing), the atmospheric VOCs were mainly influenced by combustions of coal, gasoline, petrochemicals, and compressed natural gas (CNG), while solvent use and biogenic sources also had minor contributions^24–26^. However, solvent use was the major source contributor reported in Pearl River Delta (PRD) region in China, where influenced by the emissions from a large number of factories (e.g., dye and shoemaking factories in Guangdong province)^27, 28^.

Fine particle and ground-level O_3_ are the causes for severe air pollutions, which pose high health risk as well^29–31^. In the recent years, the surface O_3_ level has been continuously increasing in most Chinese cities^32^. In northern region, the concentration in rural site had an increasing rate of 1.13 ± 0.01 ppbv year^−1^ from 2003 to 2015^32^, comparing with 0.87 ppbv year^−1^ from 2001 to 2007 at a background station in Hong Kong, where is located in Southern China^33^. And in the urban site, it was found that surface O_3_ increased as rate of 1.1 ± 0.5 ppbv year^−1^ in the city of Beijing between 2001–2006^34^, while in the city of Hong Kong, surface O_3_ increase as a rate 0.5–0.8 from mid 1960s to 2010^35^.

Baoji is an industrial city in the western edge of Guanzhong basin, and was in Shaanxi province in Northwestern China. The main industries include power plants, coal chemical industry, metal smelting, and coke productions^36^. The consumption of coal from industry sector is about ten millions tons annually (from the official statistic collected by Baoji Municipal Environmental Protection Bureau, not published yet). And the emissions plumes from both industry and traffic emissions can highly impact the air quality and elevate the surface O_3_ pollution to downwind regions^5, 37–39^. In summer, the mixing ratios of surface O_3_ in Baoji increased with ambient temperature and radiation. Its hour-average exceeded the China National Standard of 160 μg m^−3^ in a frequency of 9 to 14 days per month from May to August in 2016 (https://www.aqistudy.cn/). Ozone is the dominant factor in air pollution (https://www.aqistudy.cn/). Either speciation or evaluation of VOCs was sparsely conducted in Northwestern China^40^, and no any data were even reported in Baoji. A one-week simultaneous observation was used to evaluate the levels and compositions of both VOCs and O_3_ in this industrial city in summer, 2016. This study aims to investigate the impacts of VOCs emissions on the pollution in this typical industrial city. The Photochemical Assessment Monitoring Stations (PAMS) organized by United States Environmental Protection Agency (U.S.EPA) had defined 57 critical ozone precursors (VOCs_PAMS_)^26^. A total number of 57 VOCs that contributed mostly on the O_3_ formation in atmosphere were quantified^41^. Temporal variation and potential pollution sources were identified. The correlations among the levels of VOCs_PAMS_ and NO_x_ and meteorological factors contributed in the O_3_ formation at different locations were discussed.

## Results and Discussion

### Characterization of VOCs

Table 1 summarizes the mixing ratios of different classes of VOCs_PAMS_ quantified at the three sites (the mixing ratios of individual compound were listed in Table S1). Isoprene is classified separately from alkenes to evaluate its strong indication from biogenic sources. The average mixing ratios of total quantified VOCs_PAMS_ (TVOCs_PAMS_) were 48.03 ± 18.15, 17.00 ± 11.36, 17.27 ± 10.18 ppbv measured in the Weibin, Chencang and Miaogou sites, respectively. Comparing to the ambient levels in the Chinese megacities, our VOCs_PAMS_ in Weibin site were higher than those in Beijing in summer of 2005 (36.4 ± 12.1 ppbv), but close to Southern regions such as Guangzhou (40.58 ± 0.89 ppbv) and Hong Kong (45.83 ppbv) where influenced by industrial and traffic emissions in major^7, 8, 23^.

**Table 1:** Level (in ppbv) and compositions (in %) of VOCs_PAMS_ mixing ratio in Baoji.

		Alkane	%	Alkene	%	Aromatic	%	Isoprene	%	TVOCs_PAMS_	Reference
Baoji	Weibin site (urban)	14.00 ± 5.00	**29.2**	**8.84 ± 4.16**	**18.4**	**24.49 ± 11.36**	**51.0**	0.78 ± 0.28	1.62	48.03 ± 18.15	The present study
	Chencang site (suburban)	10.63 ± 6.88	**62.5**	1.81 ± 2.58	10.6	4.46 ± 3.32	26.2	0.59 ± 0.52	3.47	17.00 ± 11.36	
	Miaogou site (rural)	9.67 ± 5.40	**56.0**	1.23 ± 1.86	7.1	5.56 ± 4.32	32.2	0.95 ± 0.75	5.50	17.27 ± 10.18	
Beijing	Urban	15.50 ± 2.00	42.6	4.40 ± 1.10	12.1	8.60 ± 1.20	23.6	0.70–0.80	1.92	36.4 ± 12.1	Shao *et al*.(2009)
Guangzhou	Suburban	20.72 ± 1.43	56.0	7.49 ± 1.42	18.5	12.37 ± 2.17	30.5	1.10	2.71	40.58 ± 0.89	Zou, Y *et al*.(2015)
Hong Kong	Urban	32.67	71.3	7.05	15.4	6.11	13.3	0.25	0.55	45.83	Huang *et al*.(2015)

In general, the abundances of most VOCs_PAMS_ descended notably in the order of Weibin > Chencang > Miaogou. The composition of VOCs_PAMS_ significantly varied among the sites as well. The highest levels of aromatics and alkenes were observed in the Weibin site, with the mixing ratios of 16.6 ± 8.0 and 7.7 ± 4.5 ppbv, respectively. The high abundances can be ascribed to the existence of fossil fuel combustion sources installed in the industries nearby the site^10, 42^. Particularly, aromatics had a molar contribution of 51.0% of TVOCs_PAMS_ in the Weibin site (Table 1). Benzene, the most abundant aromatic in the samples of Weibin site, is an well-known tracer for the emissions of coal, biomass burning, and automobile^42^. Propene was the next abundant compound, followed by undecane, 2-methylhexane, toluene, and iso-pentane. This distribution was consistent with the emission profiles of metal smelt, coke production and power plants, which were located at the upwind position of the Weibin site^27^. The result also implies that the strong impacts from combustion sources such as industrial coal burning and vehicular emission. In comparison with the Weibin site, lower mixing ratio of benzene was measured in the Chencang site, C_2_-C_5_ alkenes, C_2_-C_5_ alkanes, C_10_-C_12_ alkanes and xylenes were higher than other VOCs_PAMS_, where the VOCs_PAMS_ profile represents the more dominance of traffic-related sources^10, 23^. This finding was relatable to the facts that the Chencang site was next to two highways (Lian-huo highway and Bao-han highway) and less factories nearby. At the Miaogou site, the mixing ratios of most VOCs_PAMS_ were the lowest among the three sites, with exceptions of toluene, styrene, undecane, isoprene, and p-diethylbenzene. Toluene and styrene are universal components in solvents used in manufactures^43^, while C_9_-C_12_ alkanes and isoprene are tracers for diesel exhaust and biogenic sources, respectively^10, 18^. This VOCs_PAMS_ distribution indicates that the air quality at Miaogou site was impacted by a mix of natural and anthropogenic pollution sources. Scattered small-scale paint factories presented around the site in the on-site survey, and the plants in the forests can contribute greatly on the biogenic emissions^10, 43^.

**Figure 1:**
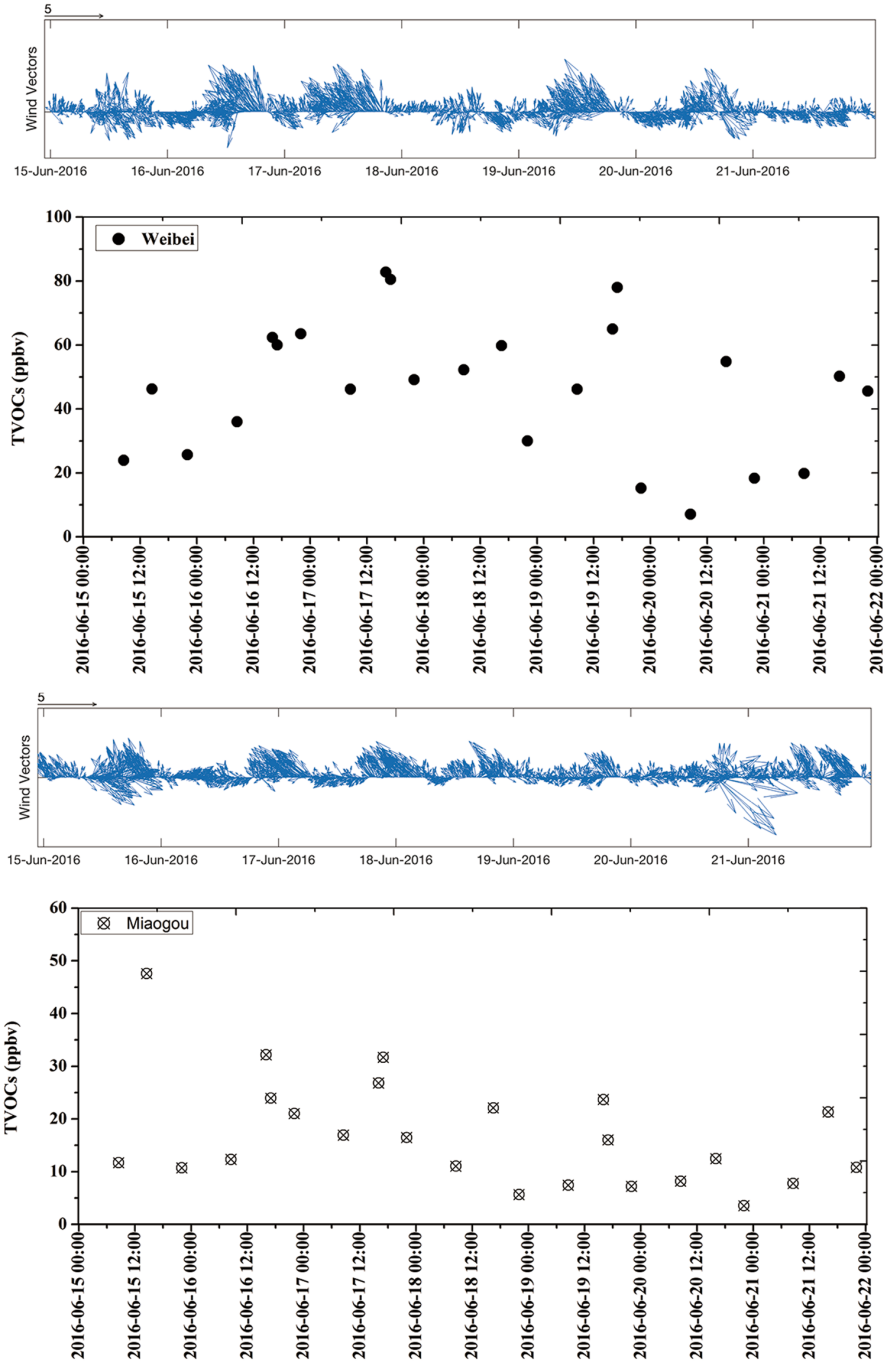
Temporal variations of TVOCs (sum of VOCs_PAMS_) with surface wind direction in Weibin site and Miaogou.

One-week temporal variation of VOCs_PAMS_ levels at the Weibin site is illustrated in Fig. 1. The mixing ratios of TVOCs_PAMS_ in Weibin site varied with surface wind directions. Higher mixing ratios of VOCs_PAMS_ were shown on the sampling days of June 16, June 17 and June 19, when the dominant wind was easterly (Figure S1). Dense power plants, metal smelt, coke production and coal chemical industries were located in the eastern and northeastern regions of Baoji. The high-frequency easterly surface winds could thus bring up those discharged pollutants to the downwind locations. The consequent effects can be also reflected on the significant elevation of corresponding marker species such as benzene and propene. In addition, from the time series, the highest TVOCs_PAMS_ was often seen in the afternoon when the easterly surface wind was dominated within a day. While the wind direction swiped from easterly to westerly gradually, the TVOCs_PAMS_ had an obvious decline. This could also demonstrate the strong impact from the industrial emissions.

### Correlation between VOCs species

**Figure 2:**
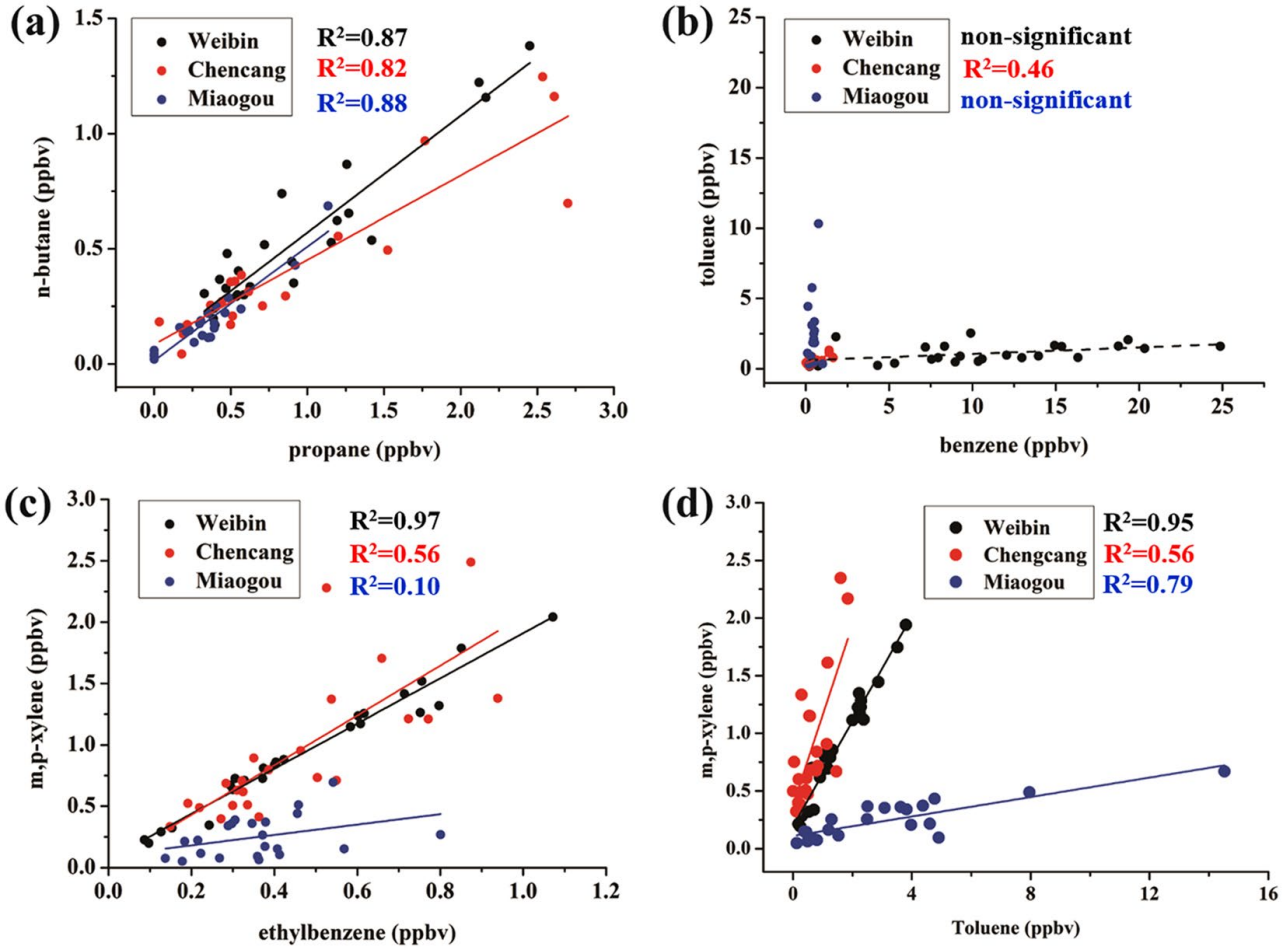
Regression plots between of VOCs species at Weibin, Chencang and Miaogou sites of Baoji.

Correlation analysis between individual VOCs_PAMS_ compound was used to interpret the potential and dominant pollution sources^31^. Propane and *n*-butane are important VOCs from vehicular emissions, and their correlation acts a useful indicator for traffic contribution^10, 31^. In the current study, propane was well-correlated with *n*-butane at both sites (0.82 < *R* ^2^ < 0.87), with slopes of 0.51, 0.37, and 0.49 for the Weibin site, Chencang and Miaogou, respectively (Fig. 2a). The ratios of *n*-butane/propane were close to that reported in a tunnel study (~0.5)^10^, demonstrating the inalienable input from vehicular emission in Baoji. The regressions of aromatics and long-chain alkanes (e.g., C_8_-C_12_) varied significantly among the sampling sites, appointing to a wide variety of source contributions (Fig. 2c, Table S2). In the Weibin site, toluene was highly correlated (*R* ^2^ = 0.50–0.95) with other aromatics (e.g., ethylbenzene, xylenes, and styrene) and *n*-decane (*R* ^2^ = 0.65) but fairly to poorly with undecane and dodecane (*R* ^2^ = 0.29 and 0.47, respectively). Reversibly, good correlations (*R* ^2^ > 0.65) between benzene and the long-chain alkanes (except *n*-decane with an *R* ^2^ = 0.22) were found. Benzene was also correlated poorly with ethylbenzene, xylenes, and styrene (*R* ^2^ = 0.24–0.29) (Table S2). Benzene is a typical VOCs_PAMS_ emitted from diesel-fueled engine, coal combustion, biomass burning, and natural gas combustion^10, 42, 44–46^, while gasoline-fueled engine emission, paint production, printing, and other solvent involved activities release more toluene, ethylbenzene, xylene and styrene in composition^10, 43^. Those correlations not only prove the high contribution of combustion sources in the Weibin site, and also suggest that the ambient levels of toluene, ethylbenzene, xylenes, styrene, 1,3,5-trimethylbenzene and *n*-decane might be additionally elevated by solvent-related industries^10, 47^. Figure 2b presents the correlations between toluene and benzene varied from Weibin site to Miaogou site. In the Weibin site, the mixing ratio of benzene was far higher than toluene (slope of toluene *verse* benzene = 0.05), ascribed to the strong benzene emissions from the local factories and transportation from the industrial zone with the easterly wind. In contrast to the Weibin site, the mixing ratios of toluene, styrene and dodecane were unexpectedly high in the Miaogou site. This also indicates that the solvent involved manufacture processes in the scattered paint and printing factories had a large contribution to the emissions of toluene and other aromatics^43^.

**Figure 3:**
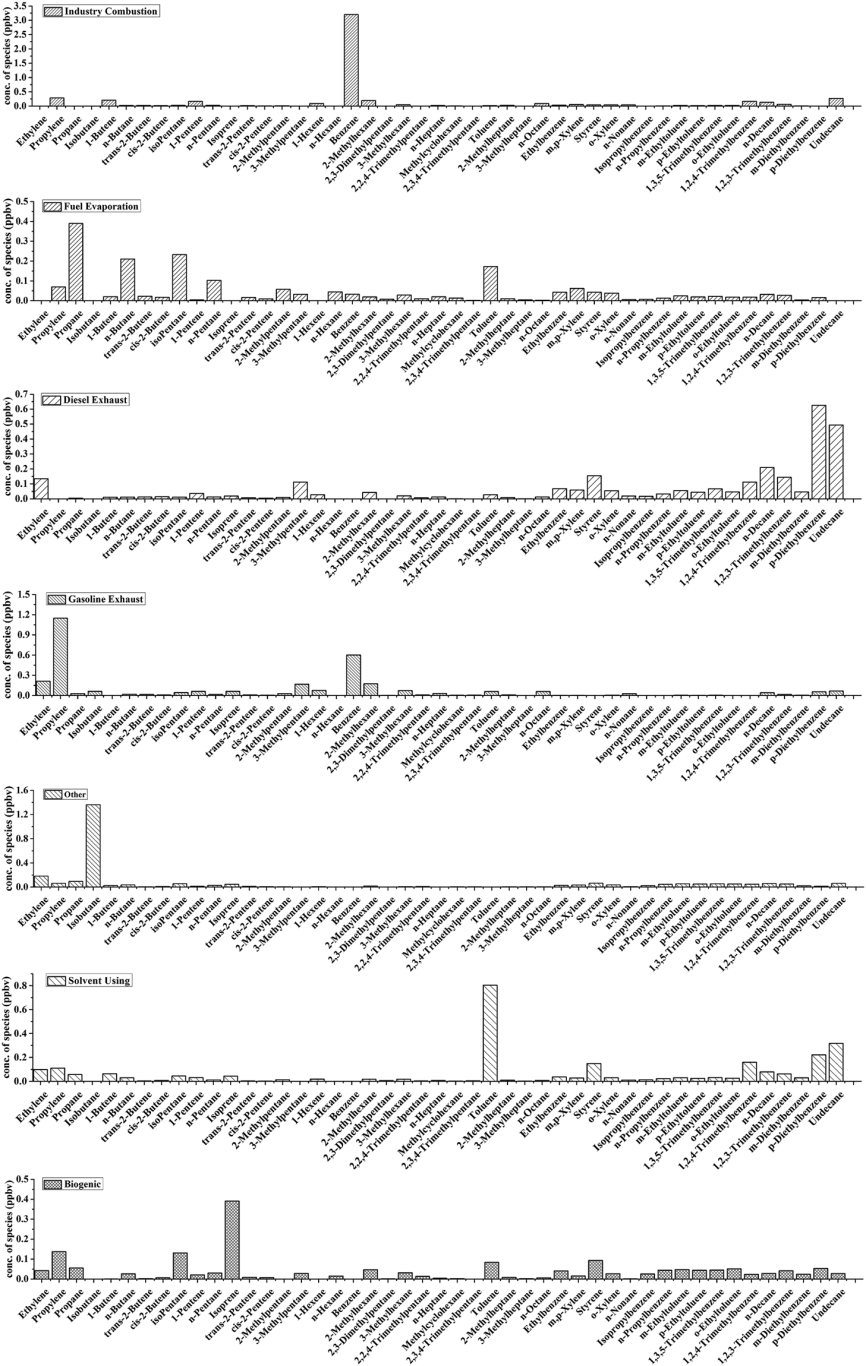
Source profiles obtained from positive matrix factorization model analysis.

The molar ratio of xylenes (*m*-/*p*-isomers) and ethylbenzene is often used to access the air mass aging, considering that the differences in degradation rates [i.e., hydroxyl (OH^•^) reaction coefficients (*K*_*OH*_)], in which xylenes have higher *K*_*OH*_ of 1.36–2.30 × 10^–11^ in comparison of lower *K*_*OH*_ of 7. 0 × 10^−12^ for ethylbenzene^31, 48, 49^. In this study, the ratios of xylenes to ethylbenzene were both high at the Weibin site and Chencang sites (1.84 and 2.03, respectively, Fig. 3d), attribute to the fresh emissions from the pollution sources. However, a much low average ratio was found in the Miaogou site (0.42), additionally with a poor correlation between the two chemicals (*R* ^2^ = 0.10). It is reasonable that relatively more aged air mass in Miaogou region due to its geographical position^48^, even though its average VOCs_PAMS_ levels were close to that at Chencang site.

### Source apportionment of VOCs_PAMS_

Source apportionment was conducted with PMF receptor model. The mixing ratios and uncertainties for the VOCs_PAMS_ from those valid samples collected in the three sites were used. Calibration was run for 3–7 factors and with random seeds. The seven factors solution produced mathematical [(Q values (both robust and true) close to the theoretical Q value] with reasonable explanation. The results are illustrated in Fig. 3. In Factor 1, it has high loadings of benzene, propylene, 1-butene and 1-pentene in a descending order in mixing ratios, that was close to the profile of coal combustion emissions^10^. Factor 2 is filled with propane, n-butane, n-pentane, iso-pentane and toluene. Most of these VOC_PAMS_ were relevant to the fuel evaporation, in particular of gasoline and CNG. Factor 3 is characterized by n-decane, undecane, xylenes, ethylbenzene, 1,2,3-trimethylbenzene, 1,2,4-trimethylbenzene, p-diethylbenzene, 3-methylpentane, ethylene, 1-pentene. The composite was corresponding to and dominated by the diesel-fuel combustion^10^. However, it must be noted that 3-methylpentane, a common marker for gasoline exhaust, was unexpectedly high in this factor. Even though the major contribution of Factor 3 could be characterized by the diesel combustion, the gasoline emission might still contribute a few. Factor 4 is consistent with a typical gasoline exhaust profile. Factor 5 is singly filled with high abundance of iso-butane but no representative source could be identified, thus is marked as others. Factor 6 is dominated by those VOCs_PAMS_ from solvent evaporation (i.e., toluene, styrene and long-chain alkanes)^43^. Isoprene is the major component in vegetation emission, even it is always detected in the vehicle exhaust^10^. Considering that high contribution of vegetation, isoprene is identified as a biogenic source marker. Factor 7 can be identified as biogenic source since isoprene had the highest contribution^10^.

**Figure 4:**
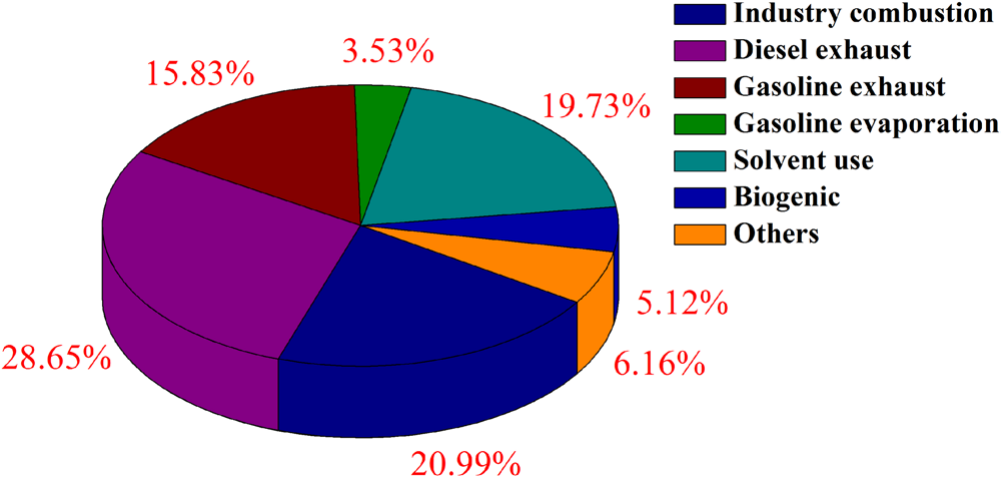
PMF derived average source contributions in Baoji.

Taking all of the samples in accounting, vehicle exhaust (44.5%), industrial discharges (20.1%), and solvent usage (19.7%) are the top three major pollution sources contributed to ambient VOCs_PAMS_ in Baoji (Fig. 4). As mentioned above, Baoji is a typical industrial city, and the main industries include power plants, coal chemical industry, metal smelting, and coke productions^36^. Most of the related industries use coal as fuel or feed, this could cause high level of benzene in ambient air. In addition, heavy industries induce dense heavy truck usage for transport in the city Baoji. These together makes high contribution of industrial and diesel vehicle exhaust.

With normalized to the mass of TVOCs_PAMS_, the contributions of the potential sources on each sample were obtained, and their strengths were compared by taking the mean values at the different sites (Table S3). The loading of TVOCs_PAMS_ in the Weibin site was ~2.8 times on average of those in the Chencang and Miaogou. The industry emission and gasoline-related sources were the two largest contributors (37.9% and 29.0%, respectively) in the Weibin site. In the Chencang, diesel exhaust was the most important source, contributing to 59.4% of the TVOCs_PAMS_. Solvent-related emission contributed 36.7% of TVOCs_PAMS_ in the Miaogou due to trace commercial painting activities. Covering with a high density of forest area, the mixing ratio of isoprene at the Miaogou was high. Therefore, a stronger strength of biogenic emission of 13.3% was found, in comparison with only 5.1 and 7.1% at the Weibin site and Chencang, respectively.

### Chemical reactivity of VOCs

The chemical reactivity of VOCs_PAMS_ was studied to investigate their potential oxidation ability with OH^•^, which can consequently lead the formation of surface O_3_^7^. The sum of OH^•^ loss rate (L_OH_) was calculated on basis of reaction rate constant between an individual VOCs_PAMS_ and OH^•^ and its mixing ratio. Table 2 lists the L_OH_ accounted at the three sites. As much higher TVOCs_PAMS_ mixing ratios at the Weibin site, higher L_OH_ (27.20 ± 12.55 s^−1^) was obtained in comparison of those at other two sampling sites. The L_OH_ in Weibin site was mainly dominated by the groups of alkenes and aromatics due to the strong influences of industrial and traffic emissions. In addition to their high chemical reactivity, the two pollution sources are thus considered to play key roles on hydroxyl radical-driven oxidations in Weibin site Baoji. The air quality at the Chencang was less impacted by the direct industrial activities, and the L_OH_ was thus less than half of that in the Weibin site. The group of alkanes, which has a lower chemical reactivity, was the major contributor (35.6%) to L_OH_. At the remote site, even though the mixing ratios of most VOCs_PAMS_ were relatively low, the high abundance of extremely-reactive isoprene dominated 32.5% to L_OH_, resulting of an equivalent level of oxidation potential as that accounted in the Chencang.

**Table 2:** Estimated OH radical loss rates with VOCs_PAMS_.

Region	L_OH_ (S^−1^)	Composition of VOCs_PAMS_ (%)			
		Alkane	Alkene	Aromatic	isoprene
Weibin site	27.20 ± 12.55	16.8	**37.7**	34.6	10.9
Chencang site	12.21 ± 6.89	**35.6**	19.6	27.1	11.2
Miaogou site	11.89 ± 8.25	**39.7**	10.8	19.6	32.5

**Figure 5:**
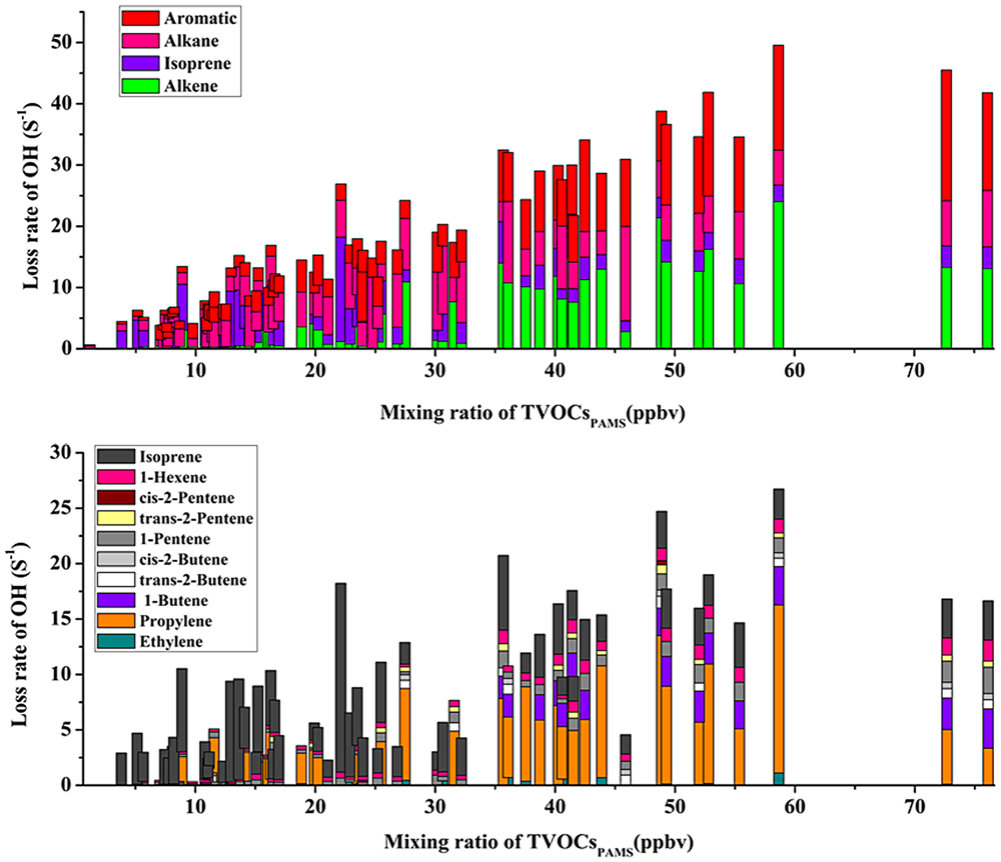
OH radicals loss rates measured with different classes of VOCs_PAMS_ (top) and abundant alkenes (C_2_-C_6_ alkenes) (bottom) in Baoji as a function of mixing ratio.

Considering that alkenes play important roles on the chemical reactivity, the contributions of the most significant individuals at different concentration levels were displaced in Fig. 5. At a low mixing ratio region (0–35 ppbv), mostly in samples collected in the Chencang and Miaogou, the biogenic-emitted compounds (i.e., isoprene) showed their dominance and strong strengths to drive the L_OH_, while the impacts from anthropogenic sources shrined as a long distance far from the two sites. At a higher mixing ratio of TVOCs_PAMS_ (>35ppbv, for the samples collected in the Weibin site), propene and 1-butene were the two most important contributors to L_OH_, representing that the industrial and traffic emissions not only can elevate the mixing ratios of VOCs_PAMS_ and amounts of OH· oxidation, and also lead further surface O_3_ formation.

### Characterization of surface O3

#### Temporal variation of surface O3

**Figure 6:**
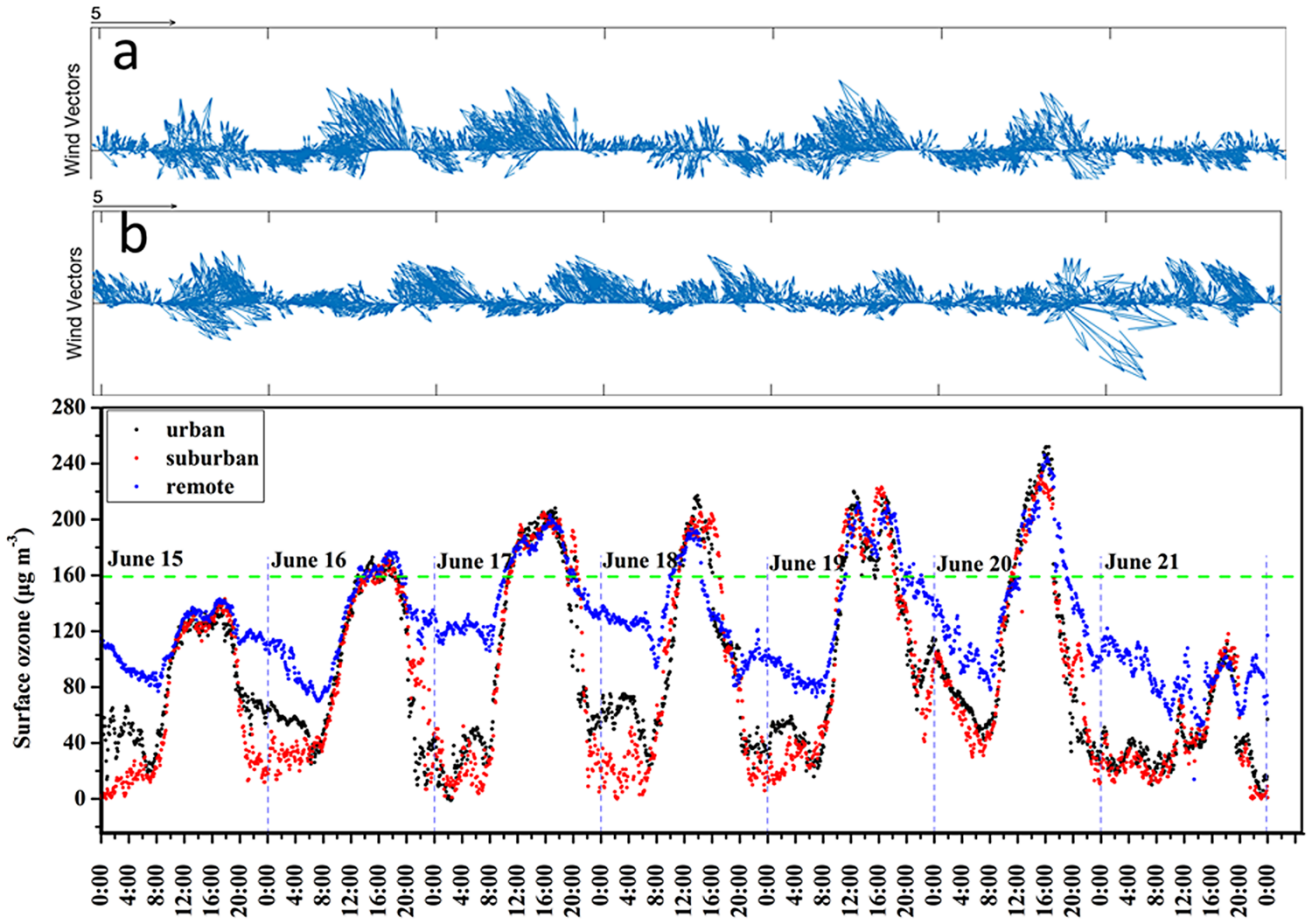
Temporal variation of surface ozone among the sampling time.

The variations of surface O_3_ at the three sites were depicted in Fig. 6. The highest O_3_ concentrations exceeded the national standard in China of 160 μg m^−3^ in the five sampling dates. The diurnal patterns of surface O_3_ levels were similar at the Weibin site and Miaogou sites, showing maxima between noon and early evening (Figure S2). In typical, double concentration peaks could be identified during this time interval. The first peak appeared around 13:00, and the next one was recorded between 16:00–17:00. The surface O_3_ concentrations decreased gradually after 18:00 and the lowest values at the Weibin site and Miaogou sites were often observed at 07:00 and 08:00, respectively. It should be noted that the surface O_3_ levels were significantly lower at the Weibin site than Miaogou site during the nighttime. The cases have been detailly explained in following sections.

#### Influences from meteorological conditions

The correlations between surface O_3_ and important meteorological parameters were fully studied. High O_3_ concentrations were often detected under high ambient temperatures and low RHs, which offer favor conditions for either formation or reservation of O_3_^50, 51^. In the current study, the O_3_ values were positively correlated to the temperature in both of the Weibin site (R^2^ = 0.59) and Miaogou (R^2^ = 0.61) during daytime, when associated with higher light intensity and radiations. Besides, water vapor could scavenge O_3_ and its precursors in atmosphere by wet deposition^52^. As expected, the surface O_3_ levels was found to be negatively correlated with RHs at Weibin site (R^2^ = 0.46) and Miaogou (R^2^ = 0.51) sites.

The O_3_ levels were also coincided with the surface zonal winds within our observation period (Fig. 6)^3, 53^. In the Weibin site, higher surface O_3_ concentrations were often seen in accordance of strong easterly wind, consistent with higher mixing ratios of TVOCs_PAMS_ measured simultaneously (Fig. 2). More obvious impacts from the direction of surface winds could be seen at the Miaogou site. Five episodes were defined between Jun 16 and Jun 20, when the O_3_ levels increased with easterly winds and under higher ambient temperatures. Particularly, the double O_3_ peaks occurred in the afternoon on Jun 16, Jun17 and Jun 19, while strong northeasterly and northerly winds were dominated. This suggested that the polluted air masses were transported from the Weibin site and industrial zone in the northeastern region of Baoji. Xue *et al*.^3^ investigated a variety of pollution origins on the formation of O_3_, and concluded that the surface O_3_ level in the Miaogou area was dominantly impacted by the exports from the well-processed industrial plumes. The results of our study were consistent with the findings. The first O_3_ peak in the afternoon was reasonably initiated by the formations from the local pollutants (i.e., photochemically reactive of isoprene emitted from biogenic sources), while the second peak could be resulted by the transportation of the industrial plume with high VOCs emission from the upwind locations.

### Ozone formation potential

While L_OH_ was used to assess the VOCs activities, ozone formation potential (OFP) could be more direct method to measure the contributions of VOCs_PAMS_ in the O_3_ formation^8, 54^. Ozone formation potential (OFP) were calculated based on the average mixing ratios and the maximum incremental reactivity coefficients (MIR) of VOCs_PAMS_ quantified in the present study^54^:

1 $${{\rm{OFP}}}_{{\rm{i}}}={{\rm{MIR}}}_{{\rm{i}}}\times {{\rm{C}}}_{{\rm{i}}}$$

where C_i_, represents the mixing ratio (ppbv) for species i. The MIRs were obtained from Carter^54^. The estimated OFP for the top 20 contributors were given in Table 3. The highest overall OFP (185.36 ppbv on average) was accounted in the Weibin site, in comparison of 42.89 and 56.74 ppbv at the Miaogou and Chencang sites, respectively. The contributions of each VOCs_PAMS_ to overall OFP varied from the sites, that might be driven by their degrees of degradation and dilution in transportation processes^48^. In the Weibin site, alkenes and aromatics were dominated and totally contributed >90% of the overall OFP loading. The marker species of propene and *m*-diethylbenzene were the two most important VOCs_PAMS_ which contributed to more than half of overall OFPs, indicating that vehicle and industrial emissions greatly affected the surface O_3_ formation. At the Miaogou site, isoprene and solvent-characterized VOCs such as toluene, styrene, xylenes, and long-chain alkanes contributed most of OFPs loading. In another aspect, air mass aging can also contribute the variation of OFPs^28^. Even though the overall estimated OFPs was low at the Miaogou site, the actual O_3_ levels were not the lowest as expected (Fig. 6). This can be ascribed to the transport of O_3_ from other upwind districts^3^. The results from the current study concluded that the fresh plume from industrial and traffic sources at the upwind location could highly impact on the air quality in Baoji, and lead the occurrence of O_3_ episodes in residential areas in summer.

**Table 3:** The top 20 most abundant O_3_ formation potential species measured at the Weibin site, Chencang and Miaogou sites of Baoji (ppbv).

Weibin site			Miaogou Site			Chencang Site		
	Average	STDEV		Average	STDEV		Average	STDEV
Alkanes	13.63	6.72	Alkanes	6.42	3.65	Alkanes	8.34	4.42
Alkenes	82.13	40.57	Alkenes	17.26	18.03	Alkenes	21.35	24.01
Aromatics	89.60	50.24	Aromatics	19.21	14.60	Aromatics	27.05	17.84
**m-diethylbenzene**	**54.67**	**39.88**	**isoprene**	**8.13**	**7.09**	**propylene**	**12.74**	**18.04**
**propylene**	**50.60**	**30.74**	**toluene**	**7.70**	**9.12**	**m,p-xylene**	**8.15**	**4.91**
1-butene	9.53	8.82	**propylene**	**4.96**	**11.51**	1,2,4-trimethylbenzene	4.04	3.31
m,p-Xylene^a^	7.35	3.84	dodecane	2.06	1.56	isoprene	3.33	4.68
isoprene	6.25	3.46	m,p-xylene	1.96	1.41	p-diethylbenzene	2.95	2.59
1-pentene	5.17	2.21	p-diethylbenzene	1.94	1.41	toluene	2.04	1.46
benzene	4.54	2.52	ethylene	1.79	3.65	dodecane	1.97	1.89
toluene	4.09	2.67	styrene	1.65	0.82	o-xylene	1.94	1.14
1,2,4-trimethylbenzene	3.56	2.16	1,2,4-trimethylbenzene	1.23	1.13	1,2,3-trimethylbenzene	1.70	1.46
p-ethyltoluene	3.25	8.55	1-pentene	1.20	0.67	ethylene	1.56	3.49
ethylene	2.48	5.22	m-diethylbenzene	0.95	1.73	1-pentene	1.53	0.93
1-hexene	2.44	1.21	ethylbenzene	0.95	0.41	1,3,5-trimethylbenzene	1.50	1.21
o-xylene	2.22	1.16	1,2,3-trimethylbenzene	0.84	0.71	ethylbenzene	1.30	0.58
isobutane	2.21	4.04	cyclopentane	0.78	1.79	styrene	1.04	0.68
2-methylhexane	1.46	0.60	o-xylene	0.76	0.52	m-ethyltoluene	0.96	0.55
cyclopentane	1.44	4.31	1-hexene	0.60	0.41	isobutane	0.83	1.61
1,2,3-trimethylbenzene	1.44	1.06	1-butene	0.58	2.78	iso-pentane	0.71	0.47
ethylbenzene	1.22	0.69	3-methylpentane	0.55	0.30	1-hexene	0.67	0.51
1,3,5-trimethylbenzene	1.17	0.70	iso-pentane	0.46	0.26	1-butene	0.65	2.92
3-methylpentane	1.15	0.63	2-methylheptane	0.46	0.56	cyclopentane	0.58	1.57

^a^ *m*-Xylene and *p*-xylene are co-eluted in the chromatographic separation.

## Conclusions

Ozone pollution control is a challenging task in China, partly due to a huge number of VOCs emissions. In this study, the high levels and compositions of alkenes and aromatics in the the ambient of Baoji suggest the large contributions from the combustion sources in surrounding industries. The temporal variations of VOCs_PAMS_ were driven by the directions of surface winds, in which the pollutants were transported from upwind regions. Aging of air mass was much obviously at the Miaogou site owing to its location distanced from the dense pollution origins. The O_3_ formation in Baoji was influenced by both export of well-processed industry plume and photochemical reactions. More long-term VOCs monitoring and environmental assessment are thus needed to interpret the characteristic roles of VOCs in the production of surface O_3_ in different regions in China. Development of mathematical models is vital to conclude the data in assistance to solve the related environmental issue.

## Material and Methods

### Description of sampling sites

Three observation fields managed by the Baoji Municipal Environmental Protection Bureau were selected and categorized as (i) Weibin, (ii) Chencang and (iii) Miaogou, respectively, in this study (Figure S3). The Weibin site (E 107°8′35″, N 34°21′17″) was located in the western part of Baoji city where is at a downwind location of an industrial zone (about 13 km). The Chencang site (E 107°14′19″, N 34° 21′44″) was located at an elementary school in the east of Baoji. No any anthropogenic pollution sources rather than vehicular emission were observed around this site. The Miaogou site (E 107°11′06″, N 34°18′23″) was set up in the southern Baoji administrative region. The location is surrounded by Qin Mountain where the forests dominated the most land covers. Very scattered factories were found nearby this region. All sampling equipment was set up on the rooftop of the site buildings, which were 10–20 m above the ground.

### Field Sampling

Samplings were conducted at 08:00–09:00, 15:00–16:00, 16:00–17:00 and 21:00–22:00, respectively, from June 15 to June 21, 2016. The VOCs in the air was drawn into a ¼” o.d. stainless steel multi-bed adsorbent tube filled with Tenax-TA, Carbograph I TD and Carboxen 1003 (C3-DXXX-5266, ca. 380 mg in adsorbent weight per tube, Markes International Ltd., Llantrisant, U.K.) using a low-flow module pump (ACTI-VOC, Markes International Ltd.) at a flow rate of 50 mL min−1 for 60 min (i.e., total sampling volume = 3 L). Insignificant breakthrough (<5%) was observed either in field or laboratory demonstration under this sampling flow and volume^52^. Two sorbent tube samples were thus collected in each time interval. The sampling inlet was set-up at 1.5 m above the ground level. Prior to the sampling, all sorbent tubes were cleaned in a thermal conditioner (TC20, Markes International Ltd.) at 330^◦^C for 20 min before use. All pre-conditioned and sampled tubes were sealed with Difflok caps (Markes International Ltd.) and stored in desiccators at 0^◦^C for a maximum of two weeks. The desiccators were filled with silica gel and activated carbon to avoid passive absorption of any water vapor and VOCs, respectively. The pump was calibrated with a mass flow calibrator (Defender 510, Bios, Torrance, CA, USA) before each sampling event. A Teflon filter assembly (47mm, Whatman, Clifton, NJ, USA) and a home-made ozone scrubber, manufactured by a 1 m long and ¼” o.d. saturated potassium iodide (KI) coated copper tube, were installed in the air upstream to remove any influences from particulate matter (PM) and O_3_, respectively. The O_3_ removal efficiency was >99% at a concentration level of 100 ppbv for 60 min in laboratory test. One field blank was collected on each sampling day. The sorbent samples were properly transported to the laboratory for chemical analysis.

Real-time concentrations of the trace gases were monitored continuously during the sampling event. NO_2_/NOx was measured with a chemiluminescence detector (Model 42i, Thermo Electron, Waltham, MA, USA), and surface O_3_ was monitored with an ultra-violet (UV) photometric O_3_ analyzer (Model 49i, Thermo Electon). The time resolution for these instruments was 5 min and the minimum detection limit (MDL) for both NO_2_/NOx and O_3_ were 0.5 ppbv. The meteorological data (i.e., temperature, wind speed/direction and Relative Humidity) were recorded with an multi-parameter automatic weather stations (WXT520, Vaisala).

### Chemical Analysis

A total of 72 valid sorbent tube samples were collected. They were all analyzed using a thermal desorption (TD) unit (Series 2 UNITY-xr system, Markes International Ltd.) coupled with a gas chromatograph/mass spectrometric detector (GC/MSD, Models 7890 A/5977B, Agilent, Santa Clara, CA, USA) within one week. The chemical analysis procedure can be found in our previous work^55^. A tube was connected into the TD unit at room temperature (~25^◦^C) and purged with ultra-high purity (UHP) helium (He) gas at a flow rate of 40 mL min−1 for 60 s to eliminate air and oxygen intrusion. For the primary desorption stage, the analytes were desorbed at 330^◦^C for 5 min and refocused onto a cryogenic-trap (U-T1703P-2S, Markes International Ltd.) to capture high volatility target compounds at −15 °C. For the secondary desorption stage, the trap was dry-purged for 6 s and rapidly heated from −15 °C to 320^◦^C and maintained for 5 min. The analytes were passed via a heated transfer line at 160 °C, and re-refocused onto a cold GC capillary column head (Rtx®-1, 105 m × 0.25mm × 1μm film thickness, Restek Corporation, Bellefonte, PA, USA) at −45 °C with an aid of liquid nitrogen (N_2_) in GC oven. Once the second desorption is completed, the oven temperature program started at an initial temperature of −45 °C for 4 min, ramped to 230 °C at a rate of 6 °C min^−1^, and maintained at 230 °C for 5 min. The constant flow rate of He carrier gas was 1.0 mL min^−1^ throughout the GC analysis. The MSD was operated in selective ion monitoring (SIM) mode at 230 °C and 70 eV for electron ionization. Identification was achieved by comparing the mass spectra and retention times of the chromatographic peaks with those authentic standards. Certified PAMS standard mixtures (Restek Corporation) were used in calibrations. A multi-point calibration curve was established to quantify each of the target compounds with linearity >0.999. The minimum detection limits (MDLs) were in the range of 0.003–0.808 ppbv with a sampling volume of 3 L. The measurement precision for the analysis of eight replicates of standard samples at 2 ppbv were ≤5%.

### Method for Source apportionment

A standard receptor model of PMF (Version 3.0) recommended by U.S.EPA was used for source apportionment in this study. In principle, the PMF model is based on the following equations:

2 $${x}_{ij}=\sum _{k=1}^{p}{g}_{ik}{f}_{kj}+{e}_{ij}$$

3 $$Q={\sum _{t=1}^{n}\sum _{j=1}^{m}[\frac{{x}_{ij}-{\sum }_{k=1}^{p}{g}_{ik}{f}_{kj}}{{\mu }_{ij}}]}^{2}$$

4 $$U=\sqrt{{(EF\times conc)}^{2}+{(MDL)}^{2}}(conc > MDL)$$

where *x*_*ij*_ is the concentration of the jth congener in the ith sample of the original data sets; *g*_*ik*_ is the contribution of the kth factor to the ith sample; *f*_*kj*_ is the fraction of the kth factor arising from congener j; *e*_ij_ is the residual between the measured X_ij_ and the predicted X_ij_ using p principal components. *μ*_ij_ is the uncertainty of the jth congener in the ith sample of the original data sets containing m congener and n samples. Q is the weighted sum of squares of differences between the PMF output and the original data sets. One of the objectives of PMF analysis is to minimize the Q value.

## Electronic supplementary material

Supporting Information
