# Supplementary material for: Source apportionment of VOCs and their impacts on surface ozone in an industry city of Baoji, Northwestern China
Source: Sci Rep. 2017 Aug 30;7:9979. doi: 10.1038/s41598-017-10631-4 (PMC5577141; doi:10.1038/s41598-017-10631-4)
Supplement: Supplementary file 1 — Supporting Information [file 41598_2017_10631_MOESM1_ESM.pdf]

Supporting Information

Source apportionment of VOCs and their impacts on surface ozone in an industry city of Baoji, Northwestern China

Yonggang Xue[1,2], Steven Sai Hang Ho[1,2,3], Yu Huang[1,2,*], Bowei Li[1,4], Liqin Wang[1,2] , Wenting Dai[1,2],

Junji Cao[1,2,*], Shuncheng Lee[5]

[1]*Key Lab of Aerosol Chemistry & Physics, Institute of Earth Environment, Chinese Academy of Sciences, Xi'an 710061, China*

[2]*State Key Lab of Loess and Quaternary Geology (SKLLQG), Institute of Earth Environment, Chinese Academy of Sciences, Xi'an 710061, China*

[3]*Division of Atmospheric Sciences, Desert Research Institute, Reno, Nevada, USA*

[4]*School of Human Settlements and Civil Engineering, Xi'an Jiaotong University, Xi'an 710049, China*

[5]*Department of Civil and Environmental Engineering, The Hong Kong Polytechnic University, Hung Hom,Hong Kong*

*Corresponding author:

Prof. Yu Huang, E-mail address: huangyu@ieecas.cn

Prof. Junji Cao, E-mail address: cao@loess.llqg.ac.cn

Tel: +86-02962336261; Fax: +86-02962336261

Supplementary Tables

Table S1. Mixing ratios of individual $VOCs_{PAMS}$ in different sampling intervals.

Compounds	Weibin Site							
	07:30-08:30		15:00-16:00		16:00-17:00		21:00-22:00	
	mean	range	mean	range	mean	range	mean	range
ethylene	0.77	bd-0.96	0.86	bd-1.82	1.13	0.19-2.92	0.20	bd-0.20
propylene	5.27	bd-8.17	6.08	2.85-9.18	7.30	4.20-12.91	5.50	bd-11.50
1-butene	1.51	bd-1.51	1.96	bd-2.53	2.18	2.01-2.48	1.61	bd-1.77
trans-2-butene	0.26	bd-0.26	0.29	bd-0.29	0.27	0.25-0.29	0.31	bd-0.37
cis-2-butene	bd[a]	bd	bd	bd	0.21	bd-0.23	0.23	bd-0.23
1-pentene	0.56	0.19-0.94	1.04	0.65-1.70	1.18	0.97-1.36	0.67	0.38-1.09
trans-2-pentene	0.17	bd-0.18	0.16	bd-0.19	0.16	0.15-0.19	0.20	bd-0.27
cis-2-pentene	bd	bd	bd	bd	bd	bd	0.13	bd-0.13
1-hexene	0.35	0.08-0.71	0.72	0.40-1.14	0.83	0.79-0.93	0.41	0.15-0.74
isoprene	0.53	bd-0.65	0.76	0.53-0.89	0.71	0.60-0.78	1.04	bd-1.50
propane	1.02	0.25-2.26	0.47	0.26-1.00	0.57	0.45-0.79	0.99	0.24-2.22
isobutane	3.81	bd-6.47	6.94	bd-12.52	9.59	bd-9.59	2.37	bd-2.90
n-butane	0.90	0.33-1.64	0.48	0.35-71	0.49	0.39-0.56	0.68	0.21-1.69
iso-pentane	0.94	0.34-1.79	0.50	0.14-0.84	0.66	0.56-0.74	0.88	0.39-1.93
n-pentane	0.58	0.26-0.99	0.38	0.23-0.64	0.44	0.34-0.49	0.54	0.32-1.08
2,2-dimethylbutane	bd	bd	bd	bd	bd	bd	bd	bd
cyclopentane	0.98	bd-0.98	8.16	bd-8.16	1.63	bd-1.63	3.03	bd-3.03
2,3-dimethylbutane	0.15	bd-0.17	0.31	bd-0.31	0.21	bd-0.21	0.09	bd-0.09
2-methylpentane	0.38	0.12-0.62	0.24	0.17-0.29	0.25	0.22-0.27	0.34	bd-0.65
3-methylpentane	0.53	0.20-0.86	0.82	0.22-1.33	1.15	0.89-1.58	0.71	0.20-1.84
n-hexane	0.28	bd-0.41	0.15	0.09-0.28	0.15	0.09-0.19	0.29	bd-0.52
methylcyclopentane	0.12	bd-0.18	bd	bd	bd	bd	0.15	bd-0.21
2,4-dimethylpentane	bd	bd	bd	bd	bd	bd	bd	bd
cyclohexane	bd	bd	bd	bd	bd	bd	0.20	bd-0.30
2-methylhexane	1.07	0.19-1.85	1.71	1.08-2.27	1.69	1.55-1.99	1.02	0.23-1.58
2,3-dimethylpentane	bd	bd	bd	bd	bd	bd	bd	bd
3-methylhexane	0.56	bd-0.81	0.65	0.40-0.77	0.58	0.57-0.58	0.82	0.11-2.77
2,2,4-trimethylpentane	bd	bd	bd	bd	bd	bd	bd	bd
n-heptane	0.24	0.17-0.31	0.27	0.20-0.37	0.28	0.25-0.30	0.24	bd-0.38
methylcyclohexane	bd	bd	bd	bd	bd	bd	0.12	bd-0.15
2,3,4-trimethylpentane	bd	bd	bd	bd	bd	bd	bd	bd
2-methylheptane	0.20	0.15-0.23	0.31	0.14-0.52	0.30	0.17-0.43	0.33	bd-0.61
3-methylheptane	bd	bd	bd	bd	bd	bd	bd	bd
n-octane	0.45	0.14-0.70	0.81	0.50-1.15	0.76	0.63-0.88	0.43	0.21-0.65
n-nonane	0.30	bd-0.44	0.60	0.31-0.93	0.52	0.43-0.60	0.31	bd-0.54
n-decane	0.67	0.08-1.65	1.36	0.34-2.47	0.78	0.22-1.46	0.63	0.13-1.39
undecane	0.43	0.08-0.65	0.89	0.29-1.25	1.01	0.70-1.16	0.37	0.10-0.72
dodecane	1.47	0.44-2.05	3.60	0.79-3.63	4.45	3.13-5.12	1.44	0.45-2.45

benzene	7.38	0.25-14.49	13.84	7.16-24.27	15.89	9.62-19.78	7.75	0.71-14.14
toluene	1.64	0.19-3.60	1.90	0.66-3.99	1.80	0.93-2.36	1.18	0.24-2.35
ethylbenzene	0.43	0.09-0.85	0.57	0.31-1.07	0.50	0.30-0.61	0.39	0.10-0.80
m,p-xylene[b]	0.89	0.23-1.79	1.16	0.73-2.04	1.01	0.63-1.24	0.71	0.20-1.32
styrene	0.38	0.02-0.83	0.64	0.19-1.41	0.62	0.27-0.86	0.29	0.03-0.81
o-xylene	0.31	0.08-0.55	0.45	0.25-0.74	0.41	0.26-0.49	0.26	0.07-0.47
iso-propylbenzene	bd	bd	0.06	bd-0.06	bd	bd	bd	bd
n-propylbenzene	0.05	bd-0.06	0.06	0.03-0.10	0.06	0.04-0.08	0.05	bd-0.06
m-ethyltoluene	0.12	bd-0.15	0.20	0.10-0.26	0.21	0.18-0.22	0.09	0.03-0.14
p-ethyltoluene	0.06	bd-0.07	0.49	0.08-2.83	4.36	0.11-7.88	0.07	bd-0.08
1,3,5-trimethylbenzene	0.08	0.02-0.14	0.17	0.08-0.25	0.17	0.11-0.22	0.08	0.02-0.13
o-ethyltoluene	0.06	bd-0.08	0.13	0.04-0.30	0.11	0.07-0.14	0.07	Bd-0.08
1,2,4-trimethylbenzene	0.28	0.05-0.43	0.60	0.27-0.79	0.63	0.45-0.76	0.24	0.06-0.45
1,2,3-trimethylbenzene	0.09	bd-0.13	0.25	0.09-0.46	0.28	0.18-0.35	0.09	0.01-0.17
m-diethylbenzene	4.34	bd-7.50	11.48	3.15-18.77	14.03	9.08-16.54	4.16	0.03-8.89
p-diethylbenzene	0.09	0.03-0.13	0.22	0.11-0.28	0.27	0.23-0.32	0.09	0.01-0.14

	Chencang site							
**Compounds**	**07:30-08:30**		**15:00-16:00**		**16:00-17:00**		**21:00-22:00**	
	**mean**	**range**	**mean**	**range**	**mean**	**range**	**mean**	**range**
ethylene	0.68	bd-1.19	1.13	bd-1.83	bd	bd	0.21	bd-0.34
propylene	4.85	bd-7.07	2.60	bd-4.67	2.06	bd-2.06	2.47	bd-2.67
1-butene	bd	bd	1.47	bd-1.47	bd	bd	bd	bd
trans-2-butene	0.25	bd-0.25	0.31	bd-0.31	bd	bd	bd	bd
cis-2-butene	0.20	bd-0.20	0.20	bd-0.21	bd	bd	bd	bd
1-pentene	0.19	0.09-0.34	0.35	0.10-0.66	0.23	0.11-0.36	0.18	0.07-0.25
trans-2-pentene	0.14	bd-0.14	bd	bd	bd	bd	bd	bd
cis-2-pentene	bd	bd	bd	bd	bd	bd	bd	bd
1-hexene	0.12	bd-0.15	0.24	0.07-0.39	0.21	0.11-0.31	0.13	bd-0.28
isoprene	0.39	bd-0.43	0.38	bd-0.48	0.44	bd-0.44	1.40	bd-2.02
propane	1.11	0.33-2.34	0.64	bd-1.04	0.39	0.38-0.40	0.76	0.07-2.43
isobutane	2.52	bd-2.97	2.04	bd-2.57	4.60	bd-4.60	bd	bd
n-butane	0.76	0.32-1.52	0.42	0.27-0.78	0.36	0.23-0.49	0.45	0.19-0.86
iso-pentane	0.76	0.28-1.03	0.43	0.18-0.62	0.31	0.20-0.42	0.49	0.05-1.26
n-pentane	0.49	0.20-0.65	0.29	0.18-0.41	0.24	0.13-0.34	0.36	0.08-0.86
2,2-dimethylbutane	bd	bd	bd	bd	bd	bd	bd	bd
cyclopentane	bd	bd	2.39	bd-2.39	1.76	bd-1.76	0.71	bd-0.71
2,3-dimethylbutane	0.14	bd-0.14	0.08	bd-0.10	bd	bd	bd	bd
2-methylpentane	0.25	0.08-0.47	0.19	0.13-0.33	0.18	0.12-0.24	0.19	bd-0.37
3-methylpentane	0.25	0.13-0.44	0.55	0.11-0.80	0.46	0.35-0.57	0.32	0.23-0.49
n-hexane	0.15	0.01-0.35	0.12	0.05-0.17	0.16	0.12-0.20	0.15	bd-0.36
methylcyclopentane	0.13	bd-0.18	0.07	bd-0.07	bd	bd	0.14	bd-0.14
2,4-dimethylpentane	bd	bd	bd	bd	bd	bd	bd	bd
cyclohexane	bd	bd	bd	bd	bd	bd	bd	bd
2-methylhexane	0.28	0.15-0.37	0.93	0.22-3.52	0.31	0.25-0.36	0.24	0.15-0.45
2,3-dimethylpentane	bd	bd	bd	bd	bd	bd	bd	bd

3-methylhexane	0.23	0.11-0.32	0.29	0.16-0.48	0.21	0.19-0.23	0.19	0.12-0.32
2,2,4-trimethylpentane	bd	bd	bd	bd	bd	bd	bd	bd
n-heptane	0.20	bd-0.24	0.18	0.12-0.23	0.13	0.12-0.14	1.20	bd-2.47
methylcyclohexane	bd	bd	0.06	bd-0.06	bd	bd	bd	bd
2,3,4-trimethylpentane	bd	bd	bd	bd	bd	bd	bd	bd
2-methylheptane	0.11	bd-0.11	0.16	bd-0.18	bd	bd	0.61	bd-1.41
3-methylheptane	bd	bd	bd	bd	bd	bd	bd	bd
n-octane	0.17	bd-0.17	0.22	0.10-0.32	0.22	bd-0.22	0.26	bd-0.28
n-nonane	0.16	bd-0.20	0.30	0.10-0.62	0.18	0.18-0.18	0.18	bd-0.30
n-decane	1.52	0.14-8.86	2.77	0.15-13.91	0.56	0.34-0.94	0.26	0.05-0.52
undecane	0.34	0.18-0.44	1.04	0.24-1.81	0.46	0.25-0.66	0.34	0.18-0.58
dodecane	3.53	0.56-9.63	9.07	0.95-18.24	3.40	2.53-4.27	3.00	0.09-5.45
benzene	0.60	bd-1.27	0.88	0.07-1.68	1.00	bd-1.00	0.93	bd-1.61
toluene	0.83	0.47-1.49	0.98	0.04-1.94	0.52	0.19-0.85	0.41	0.12-1.11
ethylbenzene	0.33	0.19-0.50	0.64	0.39-0.87	0.34	0.32-0.35	0.41	0.15-0.94
m,p-xylene	0.65	0.49-0.95	1.49	0.71-2.49	0.51	0.03-0.82	0.73	0.33-1.38
styrene	0.30	0.11-0.45	0.77	0.35-1.23	0.37	0.35-0.40	0.26	0.05-0.58
o-xylene	0.20	0.07-0.27	0.44	0.22-0.75	0.21	0.16-0.27	0.22	0.08-0.45
iso-propylbenzene	bd	bd	bd	bd	bd	bd	bd	bd
n-propylbenzene	0.04	bd-0.05	0.08	0.04-0.13	0.04	0.04-0.05	0.06	bd-0.08
m-ethyltoluene	0.09	0.05-0.14	0.19	0.09-0.31	0.11	0.10-0.11	0.10	0.04-0.21
p-ethyltoluene	0.05	0.02-0.07	0.12	0.05-0.22	0.06	0.05-0.06	0.06	bd-0.10
1,3,5-trimethylbenzene	0.07	0.03-0.13	0.26	0.07-0.51	0.14	0.14-0.14	0.09	0.02-0.17
o-ethyltoluene	0.05	0.03-0.06	0.11	0.04-0.20	0.09	0.06-0.11	0.07	0.02-0.18
1,2,4-trimethylbenzene	0.23	0.11-0.40	0.80	0.26-1.62	0.44	0.43-0.45	0.29	0.08-0.51
1,2,3-trimethylbenzene	0.09	0.04-0.16	0.33	0.09-0.72	0.21	0.18-0.23	0.12	0.03-0.25
m-diethylbenzene	0.02	bd-0.04	0.07	0.02-0.15	0.09	0.04-0.21	0.06	0.02-0.22
p-diethylbenzene	0.34	0.21-0.58	1.20	0.30-2.62	0.66	0.65-0.66	0.36	0.03-0.59

Compounds	Miaogou site							
	07:30-08:30		15:00-16:00		16:00-17:00		21:00-22:00	
	mean	range	mean	range	mean	range	mean	range
ethylene	bd	bd	1.15	bd-1.45	0.83	bd-1.08	bd	bd
propylene	2.20	bd-2.20	3.82	bd-3.82	2.30	bd-2.30	bd	bd
1-butene	bd	bd	1.50	bd-1.50	bd	bd	bd	bd
trans-2-butene	bd	bd	bd	bd	bd	bd	bd	bd
cis-2-butene	bd	bd	bd	bd	bd	bd	bd	bd
1-pentene	0.14	0.07-0.20	0.26	0.11-0.47	0.22	0.13-0.32	0.18	0.15-0.21
trans-2-pentene	bd	bd	bd	bd	bd	bd	bd	bd
cis-2-pentene	bd	bd	bd	bd	bd	bd	bd	bd
1-hexene	0.13	bd-0.14	0.19	0.10-0.24	0.17	0.11-0.24	0.15	0.07-0.23
isoprene	0.86	0.43-1.67	1.40	0.37-3.78	0.86	0.77-0.93	0.58	bd-0.74
propane	0.28	bd-0.43	0.30	0.09-0.74	0.14	0.10-0.20	0.34	bd-0.94
isobutane	bd	bd	1.79	bd-1.92	bd	bd	bd	bd
n-butane	0.29	bd-0.34	0.28	bd-0.54	0.22	bd-0.24	0.36	bd-0.74
iso-pentane	0.43	0.15-0.81	0.46	0.19-0.69	0.21	0.18-0.26	0.24	0.17-0.33

n-pentane	0.14	0.07-0.25	0.15	0.09-0.31	0.14	bd-0.15	0.17	0.05-0.34
2,2-dimethylbutane	bd	bd	bd	bd	bd	bd	bd	bd
cyclopentane	bd	bd	0.67	bd-0.87	2.80	bd-2.80	1.33	bd-2.27
2,3-dimethylbutane	bd	bd	0.27	bd-0.45	0.14	bd-0.14	0.16	bd-0.21
2-methylpentane	0.09	bd-0.10	0.11	bd-0.16	0.09	bd-0.11	0.11	bd-0.15
3-methylpentane	0.26	bd-0.39	0.55	0.27-0.85	0.44	0.37-0.52	0.26	0.10-0.47
n-hexane	0.07	bd-0.07	0.10	0.01-0.26	0.06	bd-0.07	0.04	bd-0.11
methylcyclopentane	bd	bd	bd	bd	bd	bd	bd	bd
2,4-dimethylpentane	bd	bd	bd	bd	bd	bd	bd	bd
cyclohexane	bd	bd	bd	bd	bd	bd	bd	bd
2-methylhexane	0.21	bd-0.31	0.37	0.25-0.50	0.25	0.21-0.29	0.25	0.09-0.61
2,3-dimethylpentane	bd	bd	bd	bd	bd	bd	bd	bd
3-methylhexane	0.18	bd-0.20	0.27	0.20-0.36	0.19	0.18-0.21	0.19	bd-0.26
2,2,4-trimethylpentane	bd	bd	bd	bd	bd	bd	bd	bd
n-heptane	1.98	bd-3.84	0.16	0.12-0.22	0.15	bd-0.15	0.14	bd-0.17
methylcyclohexane	bd	bd	bd	bd	bd	bd	bd	bd
2,3,4-trimethylpentane	bd	bd	bd	bd	bd	bd	bd	bd
2-methylheptane	0.44	bd-0.62	0.98	bd-2.27	0.91	bd-0.92	0.86	bd-1.71
3-methylheptane	bd	bd	bd	bd	bd	bd	bd	bd
n-octane	0.16	bd-0.16	0.19	0.15-0.25	0.17	0.16-0.20	0.19	bd-0.20
n-nonane	0.20	bd-0.20	0.19	0.14-0.25	0.17	0.15-0.19	0.17	bd-0.20
n-decane	0.30	0.05-0.71	0.73	0.16-1.51	0.64	0.40-0.90	0.33	0.09-0.88
undecane	0.25	0.11-0.59	0.86	0.19-1.14	0.85	0.60-1.22	0.33	0.08-0.68
dodecane	2.96	1.02-5.23	8.23	1.88-12.39	8.86	5.67-13.69	3.14	0.80-7.04
benzene	0.33	bd-0.51	0.64	bd-1.02	0.47	bd-0.53	0.32	0.05-0.54
toluene	2.38	0.13-4.92	4.34	0.47-15.05	2.20	0.55-4.55	2.04	0.40-4.09
ethylbenzene	0.28	0.18-0.38	0.54	0.36-0.80	0.37	0.31-0.41	0.26	0.14-0.38
m,p-xylene	0.15	0.05-0.36	0.34	0.09-0.69	0.24	0.11-0.39	0.21	0.08-0.37
styrene	0.47	0.15-0.77	1.21	0.76-1.81	0.88	0.69-0.97	0.53	0.19-0.94
o-xylene	0.08	0.05-0.16	0.18	0.06-0.32	0.12	0.07-0.17	0.10	bd-0.16
iso-propylbenzene	bd	bd	0.07	bd-0.07	bd	bd	bd	bd
n-propylbenzene	0.03	bd-0.04	0.06	0.03-0.08	0.05	0.04-0.06	0.04	bd-0.06
m-ethyltoluene	0.05	bd-0.06	0.08	0.05-0.11	0.06	0.04-0.09	0.05	bd-0.06
p-ethyltoluene	bd	bd	0.04	0.03-0.06	0.04	bd-0.04	0.03	bd-0.03
1,3,5-trimethylbenzene	bd	bd	0.07	0.02-0.12	0.06	0.03-0.09	0.04	bd-0.07
o-ethyltoluene	0.04	bd-0.06	0.04	0.02-0.07	0.06	0.05-0.08	0.04	bd-0.09
1,2,4-trimethylbenzene	0.08	bd-0.19	0.22	0.10-0.40	0.20	0.10-0.32	0.15	bd-0.22
1,2,3-trimethylbenzene	0.04	bd-0.13	0.14	0.06-0.23	0.15	0.09-0.24	0.09	bd-0.18
m-diethylbenzene	0.02	bd-0.04	0.12	0.02-0.48	0.57	0.07-1.08	0.15	0.02-0.45
p-diethylbenzene	0.22	0.07-0.52	0.67	0.14-1.06	0.63	0.39-0.93	0.29	0.07-0.60

[a] bd represents below method detection limit;

[b] m-xylene and p-xylene are co-eluted in the chromatographic separation.

Table S2. Regression parameters of different VOCs species at the three sites.

	Weibin site		Chencang site		Miaogou site	
	R^2	Slope	R^2	Slope	R^2	Slope
**n-butane/ propane**	**0.87**	0.51	**0.82**	0.37	**0.88**	0.49
**toluene/ benzene**	-	-	**0.46**	0.40	-	-
**m,p-xylene / ethybenzne**	**0.97**	1.84	**0.56**	2.03	0.10	0.42
**toluene /m,p-xylene**	**0.95**	0.20	**0.56**	0.80	**0.79**	0.02
**toluene/**ethylbenzene	**0.95**	0.24	0.26	0.20	-	-
**toluene/**styrene	**0.90**	0.33	**0.44**	0.38	-	-
**toluene/**n-propylbenzene	**0.56**	0.02	0.31	0.03	-	-
**toluene/**m-ethyltoluene	**0.50**	0.05	**0.48**	0.09	**0.40**	0.004
**toluene/**1,3,5-trimethylbenzene	**0.62**	0.05	**0.43**	0.14	**0.62**	0.007
**toluene/**n-octane	-	-	-	-	-	-
**toluene/**n-nonane	-	-	0.36	0.15	-	-
**toluene/**n-decane	**0.65**	0.54	-	-	**0.76**	0.11
**toluene/**undecane	0.47	0.25	**0.43**	0.54	**0.36**	0.07
**toluene/**dodecane	0.29	0.82	**0.48**	5.96	**0.45**	0.81
**benzene /**ethylbenzene	0.24	0.02	-	-	**0.65**	0.39
**benzene /**xylene	0.24	0.04	**0.40**	0.70	0.22	0.32
**benzene /**styrene	0.27	0.03	0.19	0.25	**0.66**	1.00
**benzene /**n-propylbenzene	0.29	0.00	-	-	**0.51**	0.05
**benzene /**m-ethyltoluene	**0.55**	0.01	0.30	0.08	-	-
**benzene /**1,3,5-trimethylbenzene	**0.54**	0.01	0.29	0.12	-	-
**benzene /**n-octane	**0.84**	0.04	**0.43**	0.07	-	-
**benzene /**n-nonane	**0.72**	0.03	0.38	0.15	-	-
**benzene /**n-decane	0.22	0.05	0.37	0.59	0.18	0.77
**enzene /**undecane	**0.65**	0.05	0.25	0.46	-	-
**benzene /**dodecane	**0.68**	0.22	0.37	6.05	-	-

Table S3 PMF derived average source concentrations and contributions at the sites.

	Weibin site		Chencang site		Miaogou site	
	mixing ratio (ppbv)	%	mixing ratio (ppbv)	%	mixing ratio (ppbv)	%
**Industry**	**17.37±13.53**	**37.9±20.8**	0.76±1.54	2.8±6.9	0.16±0.73	0.2±3.8
**Fuel Evaporation**	1.69±1.69	5.2±5.7	1.47±1.47	6.8±6.6	0.11±0.34	0.6±2.7
**Diesel exhaust**	1.31±2.59	3.0±6.8	**17.99±15.98**	**59.4±21.8**	**8.19±7.34**	**34.6±23.7**
**Gasoline exhaust**	**11.06±10.15**	**29.0±23.3**	1.59±2.32	4.5±9.7	1.40±2.26	6.2±11.7
**Painting**	4.50±5.32	10.8±15.4	1.92±3.07	6.1±12.2	**11.44±14.18**	**36.7±31.4**
**Biogenetic**	1.64±1.39	5.1±6.8	1.04±1.55	7.1±13.4	**1.99±1.30**	**13.3±13.8**
**Others**	2.46±2.08	9.0±12.7	2.17±1.91	13.3±15.1	1.04±1.34	8.4±12.8

Supplementary figures

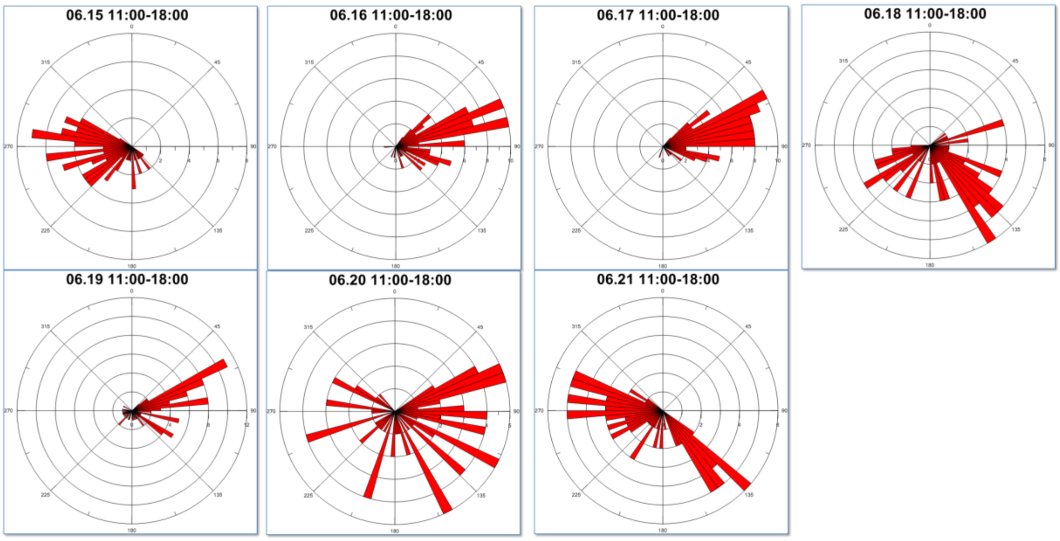

Figure S1. Wind rose diagrams for the frequencies of wind direction between 11:00-18:00 in Miaogou site.

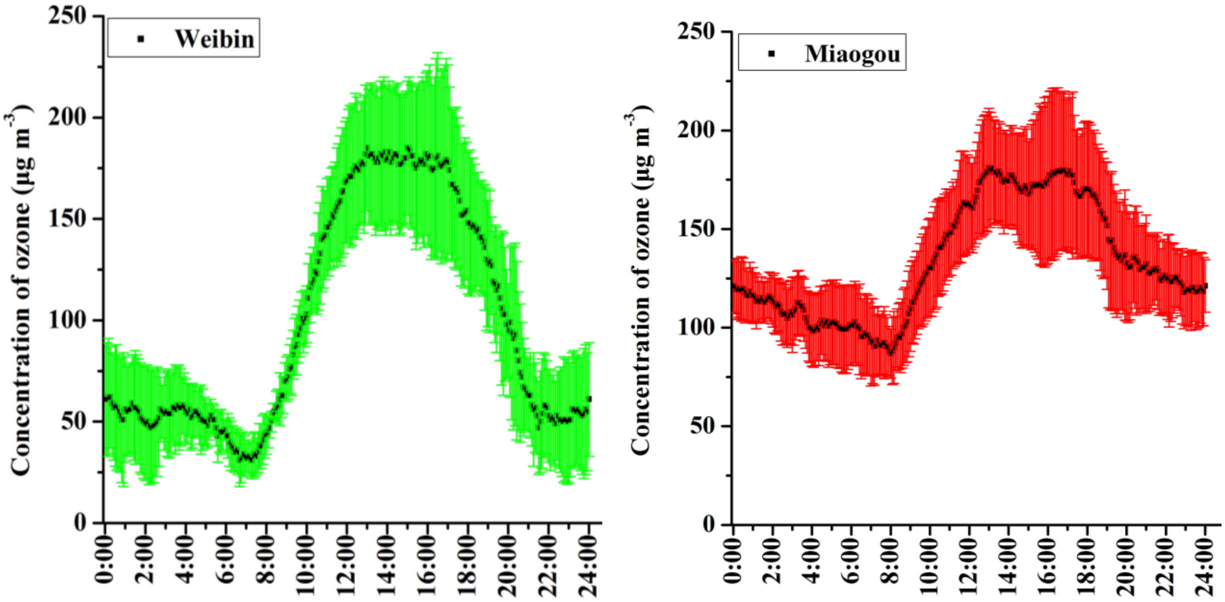

Figure S2. Diurnal variation of surface O_3 concentrations in Baoji from June 15 to June 21, 2016.

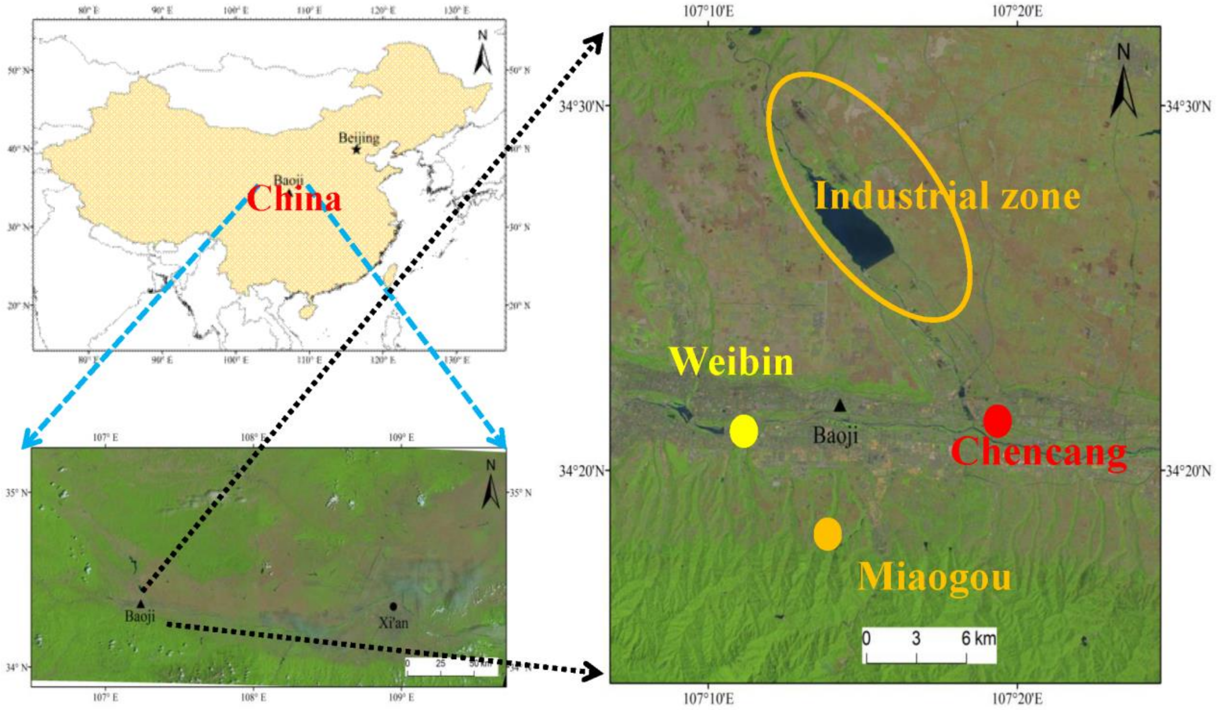

Figure S3. Sampling site of this study: Baoji is located in Northwestern China, and on the west edge of Guanzhong plain that surrounded by Qin Mountain. This map was produced with ArcGIS 10.2, software (http://www.esri.com/software/arcgis/arcgis-for-desktop), the satellite images were freely downloaded at URL: https://landsat.usgs.gov (NASA Landsat Program, 2013, Landsat8, OLI+scene LC08_L1TP_128036_20150724_20170406_01_T1, and LC08 _L1TP _127036_20150701_20170407_01_T1 SLC-Off, USGS, Sioux Falls, 07/01/2015 to 07/24/2015).
